# Genome-Wide Identification and Functional Prediction of Novel Drought-Responsive lncRNAs in *Pyrus betulifolia*

**DOI:** 10.3390/genes9060311

**Published:** 2018-06-20

**Authors:** Jinxing Wang, Jing Lin, Jialiang Kan, Hong Wang, Xiaogang Li, Qingsong Yang, Hui Li, Youhong Chang

**Affiliations:** Jiangsu Key Laboratory for Horticultural Crop Genetic Improvement, Institute of Pomology, Jiangsu Academy of Agricultural Sciences, Nanjing 200014, China; xing521_zi@163.com (J.W.); 2014104011@njau.edu.cn (J.K.); wh811006@163.com (H.W.); xiaogangli@aliyun.com (X.L.); yng_23@163.com (Q.Y.); lihui7904@163.com (H.L.); cyh@jaas.ac.cn (Y.C.)

**Keywords:** *Pyrus betulifolia*, drought stress, lncRNA, identification

## Abstract

Increasing evidence shows that long noncoding RNAs (lncRNAs) play important roles in developmental regulation and many other biological processes in plants. However, identification of lncRNAs in *Pyrus betulifolia* is limited compared with studies of functional gene expression. Using high-throughput sequencing technology, the transcriptome of *P*. *betulifolia* under drought stress was analyzed to identify lncRNAs. A total of 14,478 lncRNAs were identified, of which 251 were found to be drought-responsive. The putative target genes of these differentially expressed lncRNAs were significantly enriched in metabolic processes, organic substance metabolic processes, macromolecule metabolic processes, and heterocyclic compound binding. Real-time quantitative polymerase chain reaction validation suggested that the results of the RNA sequencing data analysis were reliable. This study will provide genetic resources for pear breeding and provide reference to other pomological studies.

## 1. Introduction

Pear (*Pyrus* spp. L) is one of the most important and popular fruit crops cultivated commercially in most temperate regions [1] and belongs to the Pomaceae subfamily of Rosaceae. The pear is a delicate fruit with a pleasant taste and smooth texture, and thus has high customer acceptance [2]. Drought is a major abiotic stress that affects pear growth, development, productivity, and quality. To improve crop performance and productivity in water-limited regions of the world, numerous studies have been conducted on drought-responsive transcriptomes and regulatory networks in several fruit trees, such as apple [3,4], cherry [5], grape [6,7], lychee [8], and pear [9].

Long noncoding RNAs (lncRNAs) are a class of RNA transcripts that do not encode proteins [10]. They are defined as more than 200 nucleotides in length with capped 5’ ends and often contain spliced introns [11,12]. lncRNAs have increasingly been shown to function as important regulatory molecules, playing many critical roles in cis- and trans-regulation of genes. lncRNAs operate transcriptionally or post-transcriptionally through diverse mechanisms, including the recruitment of factors that modify chromatin or activate transcription, serving as precursors of small RNAs and affecting nuclear architecture [13]. In humans, the expression of long intergenic noncoding RNA (lincRNA) is remarkably tissue-specific compared with coding RNA, and these lincRNAs are usually coexpressed with neighboring genes [14]. During mouse postnatal testis development, there were numerous lncRNAs overlapped or were adjacent to key transcription factors or other genes related to spermatogenesis, and most of the differentially expressed lncRNAs showed epigenetic modification marks identical with protein-coding genes [15]. lncRNAs also play vital roles in development and the stress response in plants [16,17,18]. A lncRNA was found to be involved in the NSR-ASCO-lncRNA regulatory module that regulates the alternative splicing patterns of several target mRNAs during root development in *Arabidopsis thaliana* [19]. In maize, 664 drought-responsive lncRNAs have been identified [20].

In order to identify novel lncRNAs, several strategies have been employed, including both computational and experimental screening methods [21,22]. With the development of next-generation sequencing, genome-wide identification of lncRNAs has been conducted for some plants, including *A*. *thaliana* [23], *Medicago truncatula* [24], *Gossypium* spp. [25], *Triticum aestivum* [26], *Oryza sativa* [22], and *Zea mays* [27,28]. For example, 20,163 putative lncRNAs were identified in maize using whole genome sequence annotation and RNA sequencing (RNA-seq) datasets from 30 different experiments, and more than 90% are predicted to be precursors of small RNAs. In *A*. *thaliana*, 6480 lincRNAs have been identified using bioinformatics [29], and in cotton, 50,566 lincRNAs were obtained by integrating high-quality RNA-seq data with high-depth stranded RNA sequencing [30].

Pears are reproduced primarily through grafting. As one of the main pear rootstocks, *Pyrus betulifolia* Bunge exhibits both drought resistance and salt tolerance [31,32] and identification of the drought resistance genes of *P*. *betulifolia* will be important for improving the drought resistance abilities of pears. The genome of the pear (*Pyrus bretschneideri* Rehd.) has been sequenced recently [1], but no systematic identification of lncRNAs and their responses to drought stress has been reported in *P*. *betulifolia*. In this study, RNA-seq was used to analyze differentially expressed lncRNAs during drought stress. Identification of drought-resistance lncRNA genes will be beneficial for pear breeding programs.

## 2. Materials and Methods

### 2.1. Plant Materials and Dehydration Treatment

Birch-leaf pear (*P*. *betulifolia* Bunge) seedlings were grown in the seedling beds of the Institute of Pomology at Jiangsu Academy of Agricultural Sciences, Nanjing, Jiangsu, China. Seedlings were placed in a growth chamber under a 24-h cycle: 14 h at 25 °C in the light and 10 h at 20 °C in the dark, as per our previous report [33]. Uniform and healthy six-leaf stage seedlings were inserted into a beaker containing distilled water for 2 d before dehydration treatment. Seedlings were then transferred into ½ strength Murashige and Skoog Basal (MS) solution containing 15% polyethylene glycol (PEG) to simulate drought stress. The leaves of seedlings were collected in triplicate at 0, 24, and 48 h after treatment, rinsed with distilled water, frozen in liquid nitrogen, and stored at −80 °C until further use.

### 2.2. RNA Extraction, Library Construction, and lncRNA Sequencing

To construct RNA libraries, leaves were collected after 0, 24, and 48 h of PEG- treatment of six-leaf stage seedlings, using three biological replicates. Total RNA was extracted from these samples using the Total RNA Kit (Tiangen, Beijing, China) according to the manufacturer’s instructions. RNA degradation and contamination was monitored using 1% agarose gels. RNA integrity was assessed using the RNA Nano 6000 Assay Kit with the Bioanalyzer 2100 system (Agilent Technologies, Santa Clara, CA, USA). Because some lncRNAs lack a poly(A) tail, total RNA was treated to remove ribosomal RNA (rRNA) using the Epicentre Ribo-zero rRNA Removal Kit (Epicentre, Madison, WI, USA). Finally, sequencing libraries were generated using rRNA-depleted RNA with the NEBNext Ultra^TM^ Directional RNA LibraryPrep Kit for Illumina (NEB, Ipswich, MA, USA) according to the manufacturer’s recommendations. Nine strand-specific RNA libraries were submitted to the Novogene (Beijing, China) for 150 base pair (bp) paired-end sequencing on the Illumina HiSeq 4000 platform (Illumina, San Diego, CA, USA), at a depth of 120 million reads per library.

### 2.3. Data Analysis

#### 2.3.1. Quality Control, Transcriptome Assembly and lncRNAs Identification

After removing reads that included adapter sequences or poly-*N* and low-quality sequences (Q20 < 20) from the raw data, clean data were obtained for further analysis. A reference genome and gene annotation files were downloaded from the pear genome website (http://peargenome.njau.edu.cn/). The index of the reference genome was built using Bowtie v2.0.6 [34] and clean reads were aligned to the pear genome using TopHat [35]. Based on these results, reads mapped from each sample were assembled using Cufflinks (v2.1.1) [36] with a reference-based approach.

To identify the noncoding transcripts, we used CNCI (Coding-Non-Coding-Index) [37], CPC (Coding Potential Calculator) [38], PfamScan [39] and PhyloCSF [40] software to distinguish protein coding and non-coding sequences. If a sequence was predicted to be potential coding by at least one of the tool, it was considered as a potentially coding or a pseudogene and excluded from the final dataset. Resulting sequences smaller than 200 nt were considered small noncoding RNA, while those bigger or equal than 200 nt were considered long noncoding RNA. We filtered out the low expressed transcripts and retained the transcripts characterized by FPKM (fragments per kilobase of exon per million fragments mapped) value ≥0.5 using Cuffquant [41].

To identify lncRNAs acting as precursors of miRNA in birch-leaf pear, the lncRNAs were aligned with the precursors of known microRNA (miRNAs) in the miRNA Base 21.0 (http://www.mirbase.org/) using BLAST with default parameters [42]. The lncRNAs homologous to miRNA with >90% coverage were eventually defined as miRNA precursors. Based on multiple sequence alignments, secondary structures, and covariance models, all predicted lncRNAs were classified into families that shared a common evolutionary ancestor using Rfam [43] and INFERNAL [44] with default parameters.

#### 2.3.2. Differential Expression Analysis

The expression levels of every isoform were calculated using the FPKM method, which is calculated based on the lengths of the fragments and the read count mapped to a particular fragment. We used the Cuffdiff (v2.1.1) software [45] to calculate FPKMs of the lncRNA genes in each sample. A *p*-value was assigned to each gene and adjusted by the Benjamini and Hochberg approach for controlling the false discovery rate [46]. lncRNAs with an adjusted *p*-value < 0.05 were set as the threshold for significant differential expression [47].

#### 2.3.3. Putative Target Gene Prediction and Enrichment Analysis

To predict the putative target genes of significantly differentially expressed lncRNAs two approaches were used, coexpression and colocation method. Coexpression were predicted based on the expression correlation coefficient (Pearson’s correlation > 0.95 or < −0.95). In colocation, we searched for all the protein-coding genes 10 kb upstream or downstream of the differently expressed lncRNAs [48]. Gene ontology (GO) and Kyoto Encyclopedia of Genes and Genomes (KEGG) analyses of the putative target genes were conducted respectively. GO enrichment analysis was conducted using the GOseq R package v1.32, and GO terms with corrected *p*-values less than 0.05 were considered significantly enriched. KEGG orthology based annotation system (KOBAS) software was used to test for statistical significance of the differentially expressed lncRNA putative target genes in KEGG pathways (http://www.genome.jp/kegg) [49].

#### 2.3.4. Quantitative Real-Time Polymerase Chain Reaction Analysis

The expression profiles of drought-responsive lncRNAs were validated through quantitative PCR. Total RNA for use as a template was extracted from leaves using the Total RNA Kit (TIANGEN) according to the manufacturer’s instructions, the first cDNA strand was synthesized from 1000 ng total RNA in a volume of 20 μL using the PrimeScript RT Reagent Kit with genomic DNA (gDNA) eraser (Perfect Real Time), (Clontech, Shiga, Japan), according to the manufacturer’s protocol. Twelve lncRNAs were randomly selected from the differentially expressed lncRNA and analyzed using Real-Time Quantitative Polymerase Chain Reaction (RT-qPCR). Primers were designed using Primer Premier 5.0 software [50], and RT-qPCR was performed on a 7500 Real-Time PCR System (Applied Biosystems, Carlsbad, CA, USA). The total reaction volume was 20 μL, containing 10 μL 2× SYBR Premix Ex Ta (TaKaRa Bio Inc., Shiga, Japan), 1 μL complementary DNA (cDNA) reaction mixture, 0.5 μL of each primer with concentration is 10 pmol/μL, 0.5 μL ROX Reference DyeII (50×), and 7.5 μL ddH_2_O. The primers sequence used in our PCR experiments are described in Supplementary Table S11. PCR was performed as follows: pre-denaturation at 95 °C for 30 s, denaturation at 95 °C for 3 s, annealing at 60 °C for 30 s, and 55–95 °C for melting curve analysis. All reactions were performed using biological triplicates. The 2^−ΔΔ*C*t^ method was used to calculate relative changes in gene expression between control and treatment plants [51]. Ubiquitin (*UBQ*) gene was used as housekeeping gene for normalization [52].

## 3. Results

### 3.1. Identification of lncRNAs in the Birch-Leaf Pear

To investigate novel and drought-responsive lncRNAs in *P*. *betulifolia*, we performed Illumina sequencing of RNA from the PEG-stressed pear using Hiseq2500. In total, we obtained 877.97 million raw reads from nine samples. After filtering out adapter sequences and low-quality reads, we obtained 853.77 million clean reads for further analysis (Table 1).

**Figure 1:**
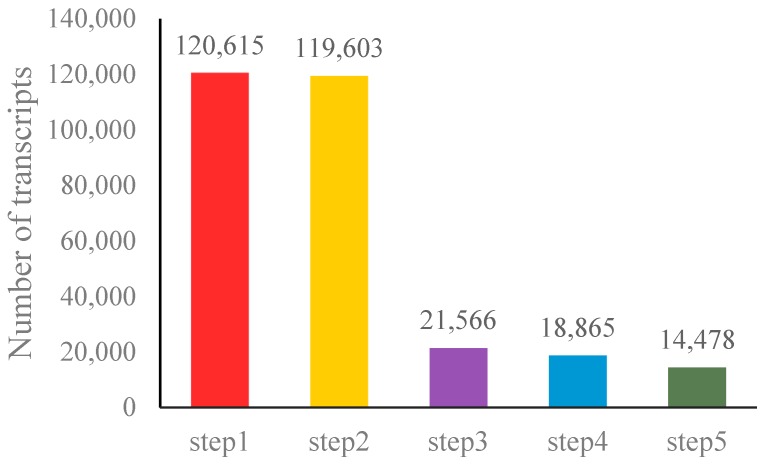
Statistics of candidate long noncoding RNAs (lncRNAs). Five filter processes were used to identify lncRNAs. Step1 filtered out the low expression transcripts. Step2 filtered transcripts with length <200 nucleotides. Step3 filtered out the transcripts that contain exon region. Step4 selected transcripts with FPKM (fragments per kilobase of exon per million fragments mapped) ≥0.5. In Step5 we selected for coding potential, and finally 14,478 lncRNAs remained.

Sequence reads were mapped with TopHat [35] and assembled with Cufflinks [45]. We discovered 120,615 total transcripts. To identify pear lncRNA candidates in these 120,615 transcripts, the transcripts were further analyzed using computational and experimental methods. Five sequential stringent filter processes were used to identify lncRNAs (Figure 1). First, we filtered out transcripts with low expression and low-credibility single transcripts, and selected sequences with exon number ≥2. Second, we filtered transcripts with length <200 nucleotides. Three, using Cuffcompare software, we filtered out the transcripts that overlap with the database annotation exon region. Fourth, we filtered by expression, selecting transcripts with expression FPKM ≥0.5. Fifth, we selected for coding potential, and finally obtained 14,478 reliably expressed novel lncRNAs (Supplementary File S1), including 10,896 lincRNAs and 3582 antisense lncRNAs.

### 3.2. Characterization of Pear lncRNAs

**Figure 2:**
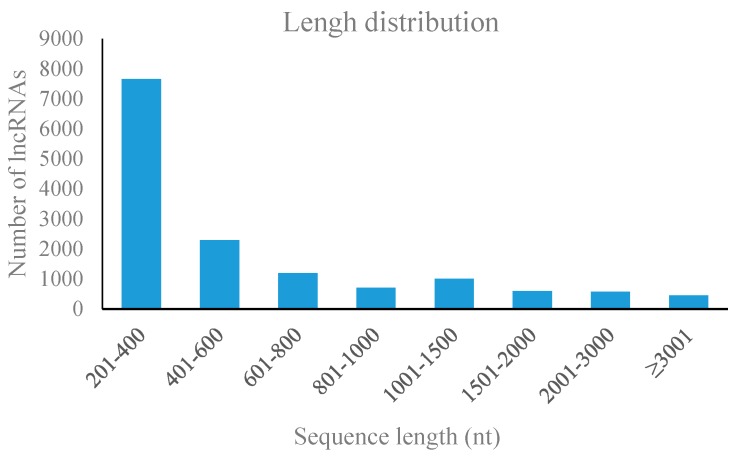
Length distribution of the 14,478 pear lncRNAs.

Based on the results, 14,478 putative lncRNAs were selected and used for further analysis. The lncRNAs ranged in length from 200 to 9334 bp. The most abundant length was 200–400 bp (Figure 2). In addition, the lncRNAs distribution in pear genome was examined. There was an average of 30.4 lncRNAs per Mbp. The eighth chromosome had the highest lncRNA packing density and per Mbp nucleotides contained 41.9 lncRNAs. The lowest packing density located on the fifth chromosome and containing 17.2 lncRNAs per Mbp of nucleotides. By classify the lncRNAs into different non-coding RNA (ncRNA) families, the structure, properties and functions of these predicted lncRNAs can be better annotated and understood. All lncRNA candidate were divided into different ncRNA families using INFERNAL [44]. Global detection of the lncRNAs sequences and secondary structures indicated that most of the pear lncRNAs belong to seven conserved ncRNA families: 5SrRNA, transfer RNA (tRNA9, snoR, SNORD and mir (Supplementary Table S2 F). In total, 77 lncRNAs were detected as precursors for the 240 miRNAs, for example, five miRNA precursors (gma-MIR393c, gma-MIR393d, gma-MIR393e, gma-MIR393f and gma-MIR393g) could be aligned with lncRNA3103. The miRNA precursors are shown in Supplementary Table S3.

### 3.3. Identification of lncRNAs that Respond to Drought-Stress Treatments

**Figure 3:**
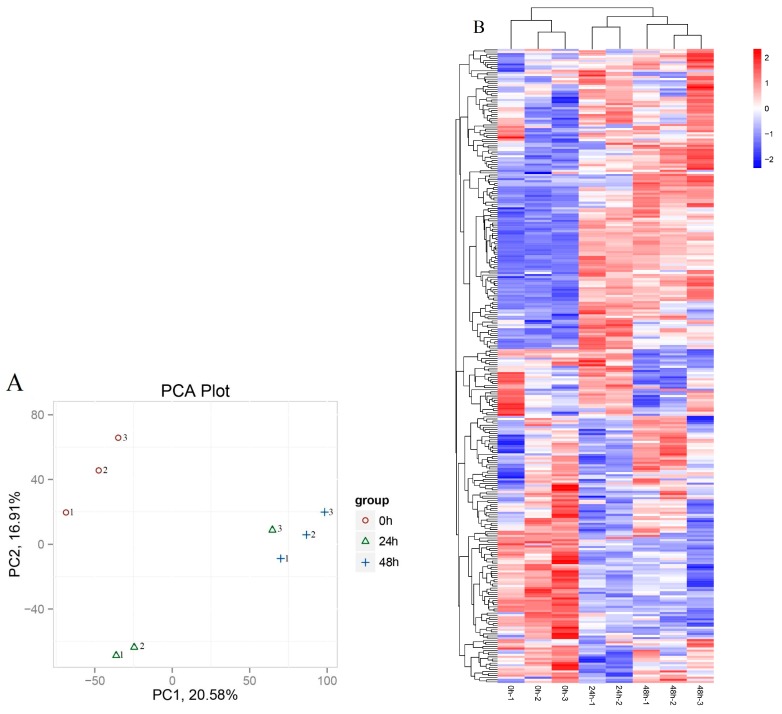
Principal component analysis (PCA) and heat map of lncRNAs during the different drought stages. (**A**) PCA of lncRNAs during different drought stage. x-axis is the contribution rate of the first principal component to the sample difference, y-axis is the contribution rate of the second principal component to the sample difference. 0 h and 48 h samples clustered together, but h24–3 does not cluster with its replicates; (**B**) A hierarchical clustered heat map showing the log_10_ transformed expression values for differently expressed lncRNAs between 0 h, 24 h and 48 h. Red represents higher expression and blue represents lower expression. 0 h, 24 h and 48 h indicate that the samples were treated under drought stress for 0 h, 24 h and 48 h, respectively.

To identify which of the 14,478 lncRNAs were significantly differentially expressed under drought stress treatments, the sequences were sorted using fold-change values of FPKM expression, then filtered based on false discovery rate ≤0.05 and log_2_ ≥1. The principal component analysis (PCA) showed the reproducibility of the lncRNA expression, the h24–3 sample do not cluster with its h24–1 and h24–2 replicates (Figure 3A), so it was excluded to avoid tampering posterior expression analyses. The results indicated that the expression of 251 lncRNAs changed significantly by take the 24 h and 48 h expression values compared to 0 h (Supplementary Table S4). We identified 191 lncRNAs with significantly different expression between the 0 h and the 24 h, of which 92 genes were upregulated and 99 were downregulated. A total of 126 lncRNAs exhibited significant differential expression between 0 and 48 h, with 72 genes upregulated and 54 downregulated. The greatest number of differentially expressed transcripts was between 0 h and 24 h. The differentially expressed lncRNAs among the three stages are represented in a heat map (Figure 3B).

Comparison of changes in lncRNAs between the two groups uncovered similarities and considerable differences. There are 125 unique significantly differentially expressed lncRNAs in the comparison of 24 h vs 0 h, 60 unique significantly differentially expressed lncRNAs in the comparison of 48 h vs 0 h. For the two groups, whereas 66 lncRNAs are common to both groups, indicating that lncRNAs are differentially expressed depending on the dehydration status of the plant.

### 3.4. Functional Prediction of Drought Response lncRNAs

**Figure 4:**
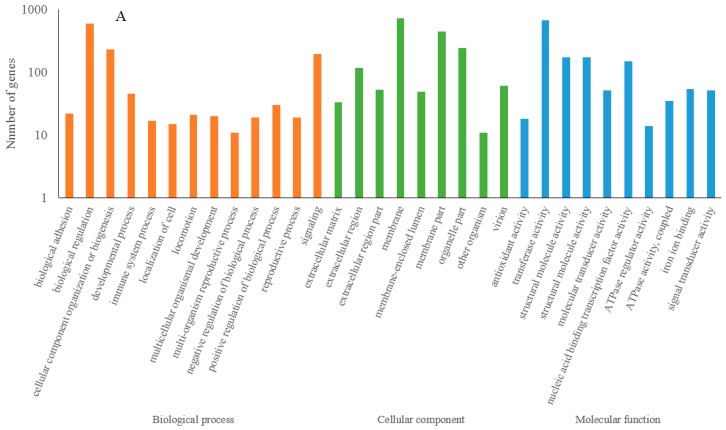

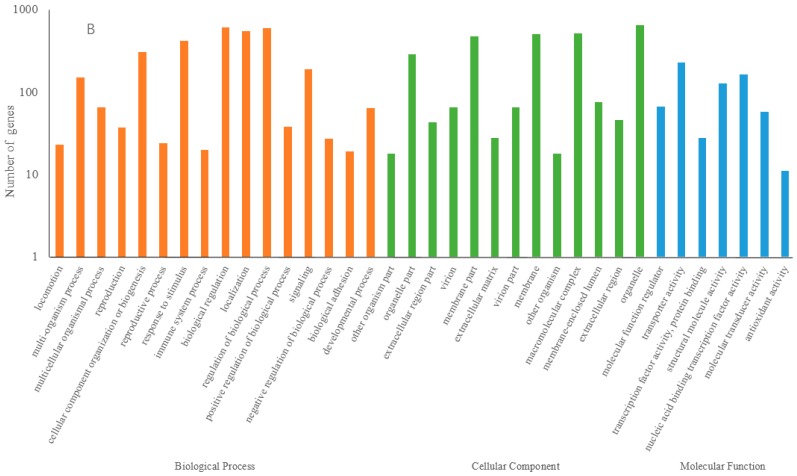
Functional analysis of differentially expressed lncRNA in drought stress. (**A**) Gene ontology (GO) analysis of putative target genes of differentially expressed lncRNA of 24 h vs. 0 h; (**B**) GO analysis of putative target genes of differentially expressed lncRNA of 48 h vs. 0 h. GO analysis contains three branches: biological process (orange), cellular component (green) and molecular function (blue). 0 h, 24 h and 48 h indicate that the samples were treated under drought stress for 0 h, 24 h and 48 h, respectively.

To understand the potential functions of lncRNAs during drought stress, we conducted GO and KEGG enrichment analysis of the putative target genes of differentially expressed lncRNAs. We first calculated the Pearson correlation coefficient between lncRNAs and messenger RNAs (mRNAs) by examining the paired lncRNA and mRNA expression profiles. For differently expressed lncRNAs and their putative target genes between 24 h and 0 h. a total of 3139 positive and 1939 negative interaction relationships putative target genes were obtained, for 48 h and 0 h a total of 3967 positive and 1486 negative interaction relationship putative target genes were detected. GO enrichment (Figure 4A,B) analysis showed that the putative target genes of differentially expressed lncRNAs were significantly enriched in metabolic processes, organic substance metabolic processes, catalytic activity, and structural molecule activity (Supplementary Tables S5 and S6). KEGG is a database for determining the high-level functions of biological systems. KEGG pathway analysis of the putative target genes revealed that the most significant pathways were ribosome, proteasome, photosynthesis and biosynthesis of amino acids Supplementary Tables S7 and S8).

Previous studies showed that lncRNAs could play a cis-regulatory role by mediating the expression of neighboring genes. We sought the 10 kb upstream or downstream of the differentially expressed lncRNAs to find protein-coding genes. We found 3010 protein-coding genes close to 185 differentially expressed lncRNAs between 24 h and 0 h, and 2158 protein-coding genes were located close to 126 differentially expressed lncRNAs between 48 h and 0 h. GO term analysis of the putative target genes were annotated as regulating biological process, response to stimulus, and catalytic activity (Supplementary Tables S9 and S10). KEGG pathway analysis of the putative target genes showed that the most significant pathways were proteasome, photosynthesis, oxidative phosphorylation and metabolic pathways (Supplementary Tables S11 and S12).

The response to drought stress in plants is a complicated process, involving several genes and metabolic network, hormones synthesis is one of important factor [53]. In total, we found that 9 lncRNAs were associated with auxin and 2 lncRNAs were associated with cytokinin. Oxidation-reduction processed pathway is another important pathway on drought stress, in this study, a total of 43 putative target genes associated with this pathway in common between the sets of co-expressed and co-located. We also identified approximately 69 putative target genes linked with ubiquitin, which may take part in signal transduction and the degradation of protein in response to stress [54] (Supplementary Tables S13 and S14).

### 3.5. Real-Time Quantitative Polymerase Chain Reaction Validation

**Figure 5:**
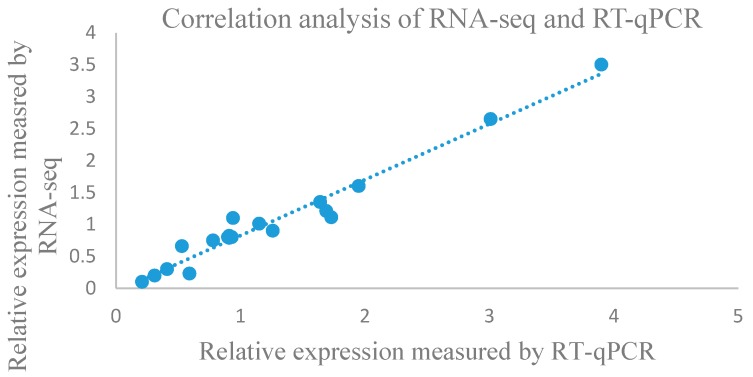
Confirmation of the expression patterns of lncRNAs using real-time quantitative polymerase chain reaction (RT-qPCR). Ten lncRNAs were randomly selected from differentially expressed lncRNA on different time point of drought stress. The *x*-axis indicates the relative expression measured by RT-qPCR. The *Y*-axis indicates relative expression measured by RNA sequencing (RNA-seq). The Pearson correlation of relative expression measured by RNA-seq and RT-qPCR was 0.91; ubiquitin (*UBQ*) was used as the reference gene.

To validate the reliability of the transcriptome gene expression profiles, 10 differentially expressed lncRNAs were randomly selected for expression analysis through RT-qPCR. The expression patterns shown in the RT-qPCR results were consistent with the RNA-seq results (Figure 5). For example, the relative expression of lncRNA7695 was increase on 24 h but decreased on 48 h—this was consistent with the RNA-seq result (Supplementary Table S15). We also validated the location of lncRNA7695 and lncRNA4073. They were located in scaffold1249.0 and scalffold215.0, respectively (Supplementary Figure S1). Both results suggest the reliability of the experiment data analysis in terms of expression data and sequence coverage.

## 4. Discussion

We obtained a large number of lncRNAs from the birch-leaf pear: 14,478 lncRNAs were reliably identified. Among other plants, 50,566, 6480, 2224, and 20,163 lncRNAs were previously identified in *Gossypium* spp. [30], *Arabidopsis* [29], rice [20], and two studies of *Zea mays* [27], respectively. The number of pear lncRNAs we identified is greater than the number identified in *Arabidopsis*, rice, but fewer than in *Gossypium* spp. and *Z. mays*. In *Gossypium* spp. and *Z. mays*, cDNA libraries were generated from multiple tissue types, whereas the cDNA libraries in the present study were generated only from leaves. Therefore, we likely identified only a subset of the total lncRNAs in birch-leaf pear, and we believe that more lncRNAs may be identified using different organs and under different stresses.

In contrast to mRNAs, lncRNAs are expressed at a low level in a tissue-specific manner, and generally exhibit poor conservation across different species [55]. lncRNAs can regulate gene expression in various ways under abiotic and biotic stresses. For example, lncRNA plays a critical role controlling the stress-responsive in *Populus* and wheat [16,56]. lncRNA173 target gene SUCROSE SYNTHASE 4 showed responsive to high temperature in *A. thaliana* [57]. In wheat, 125 lncRNAs responsive to powdery mildew infection and heat stress were identified [15]. In our result, the expression levels of 251 lncRNAs were found to change significantly during our three-stage treatment. These results indicate the drought stress damage affected the expression of some lncRNAs.

Plants are frequently affected by a wide range of environmental stressors [58]. To cope with this stress, plants have evolved physiological and biochemical mechanisms to adapt to stress [59]. To elucidate the function of putative target genes of differently expressed lncRNAs, we assigned the putative target genes of lncRNAs to various GO categories and identified several associated with the drought-stress response, such as those related to metabolic processes, organic substance metabolic processes, and catalytic activity. Using the KEGG database, we annotated the putative target genes to specific pathways, among which metabolic pathways accounted for a great proportion. In a previous study, 662 European beech genes were found to be differentially expressed under drought stress, and a large proportion of these genes were categorized into metabolic pathways [60]. Based on KEGG analysis of drought stress in Chinese fir (*Cunninghamia lanceolata*), the largest proportion of differentially expressed genes were related to metabolic pathways [61]. Thus, in this study, we obtained results consistent with those of previous research, providing further insight into the role of lncRNAs in the drought response.

In summary, we identified 14,478 lncRNAs in birch-leaf pear and showed that 251 were dehydration-responsive. These results indicate that the drought stress affected the expression of some lncRNAs. The putative target genes of different expressed lncRNA were identified, some of these putative target genes are involved in desiccation-response processes. The results of RT-qPCR validated the reliability of the RNA-seq. Our results provide a rich genetic resource for discovery of genes related to drought stress and can be readily applied to other fruit tree species.

## Figures and Tables

**Table 1:** Sequencing data for nine libraries obtained by RNA sequencing.

Sample Name	Raw Reads	Clean Reads	Clean Bases	Error Rate (%)	Q20 (%)	Q30 (%)	GC Content (%)
h0–1	104,464,354	101,335,652	15.2 G	0.01	97.61	93.56	43.19
h0–2	92,734,618	90,096,636	13.51	0.02	95.63	88.03	44.27
h0–3	89,700,722	87,193,746	13.08 G	0.03	95.00	86.72	43.49
h24–1	99,690,832	96,780,130	14.52 G	0.02	97.51	93.32	43.07
h24–2	96,641,070	94,021,570	14.1 G	0.02	97.37	93.04	43.60
h24–3	99,260,444	96,754,238	14.51 G	0.02	97.57	93.46	43.59
h48–1	102,684,392	99,947,264	14.99 G	0.02	97.57	93.48	43.02
h48–2	98,612,476	95,718,804	14.36 G	0.02	97.56	93.45	43.60
h48–3	95,021,280	92,290,956	13.84 G	0.02	97.47	93.24	43.35

Q20 and Q30 are the percentages of reads with Phred scores over than 20 and 30, respectively. GC content (%) means G + C bases as the percentage of total bases. 0 h, 24 h and 48 h represented the samples that were treated under drought stress for 0 h, 24 h and 48 h respectively.

## Supplementary Materials

The following are available online at http://www.mdpi.com/2073-4425/9/6/311/s1, File S1. The 14,478 lncRNA sequences obtained by RNA sequencing of *Pyrus betulifolia* in control and drought stressed conditions. Table S1. Sequence of the primers used in qRT-PCR analysis. Table S2. The family classify of lncRNA of *Pyrus betulifolia* obtained from drought stress condition. Table S3. lncRNA target precursors for miRNA in *Pyrus betulifolia* under drought stressed conditions. Table S4. The significantly different expressed lncRNAs between 24 h and 48 h compared to 0 h of *Pyrus betulifolia* under drought stress. Table S5. Gene Ontology (GO) of putative target genes of differentially expressed lncRNA of 24 h vs. 0 h based on coexpression. Table S6. Gene Ontology(GO) of putative target genes of differentially expressed lncRNA of 48 h vs. 0 h based on coexpression. Table S7. KEGG enrichment result of putative target genes of differentially expressed lncRNA of 24 h vs. 0 h based on coexpressionTable S8. KEGG enrichment result putative target genes of differentially expressed lncRNA of 48 h vs. 0 h based on coexpression. Table S9. Gene Ontology(GO) of putative target genes of differentially expressed lncRNA of 24 h vs. 0 h base on colocation. Table S10. Gene Ontology(GO) of putative target genes of differentially expressed lncRNA of 48 h vs. 0 h base on colocation. Table S11. KEGG enrichment result of putative target genes of differentially expressed lncRNA of 24 h vs. 0 h base on colocation. Table S12. KEGG enrichment result of putative target genes of differentially expressed lncRNA of 48 h vs. 0 h base on colocation. Table S13. lncRNA and their putative target mRNA based on the coexpression results. Table S14. lncRNA and their putative target mRNA based on the colocation results. Table S15. Relative expression data measured by qRT-PCRs and RNAseq. Figure S1. The genome browser with read coverage of the lncRNA7695 and lncRNA4073.
