# Supplementary material for: Genome-Wide Identification and Functional Prediction of Novel Drought-Responsive lncRNAs in Pyrus betulifolia
Source: Genes (Basel). 2018 Jun 20;9(6):311. doi: 10.3390/genes9060311 (PMC6027255; doi:10.3390/genes9060311)
Supplement: Supplementary file 1 [file genes-09-00311-s001.zip › Figure S1 - The genome browser with read coverage of the lncRNA7695 and lncRNA4073.docx]

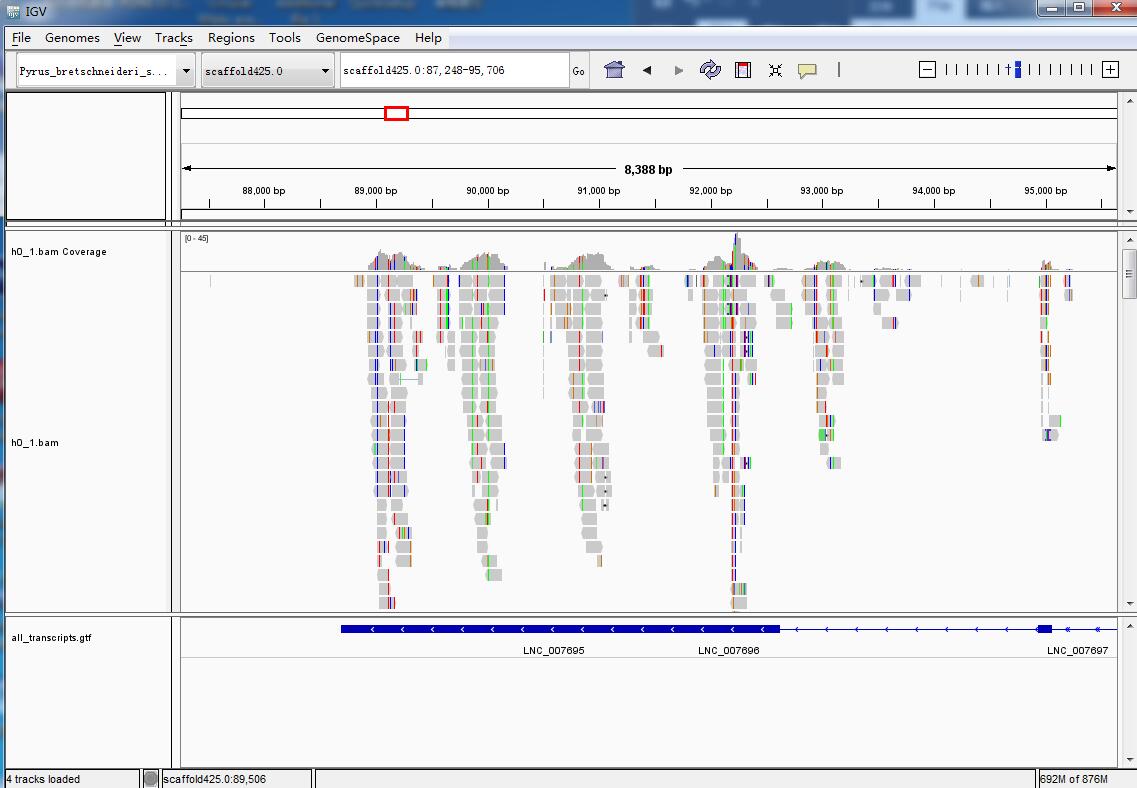


1. The genome browser with read coverage of the lncRNA7695.


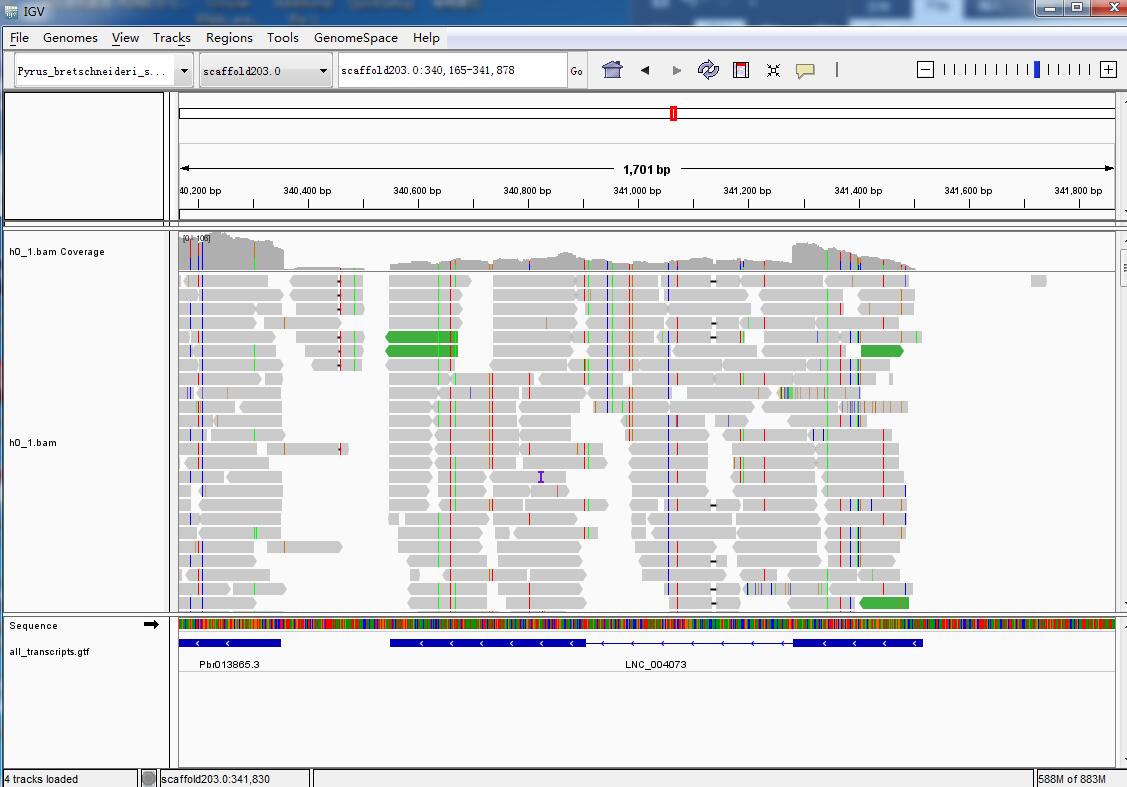


B:The genome browser with read coverage of the lncRNA4073.
