# Supplementary material for: Genome-Wide Identification and Functional Prediction of Novel Drought-Responsive lncRNAs in Pyrus betulifolia
Source: Genes (Basel). 2018 Jun 20;9(6):311. doi: 10.3390/genes9060311 (PMC6027255; doi:10.3390/genes9060311)
Supplement: Supplementary file 1 [file genes-09-00311-s001.zip › Table S1 - Sequence of the primers used in qRT-PCR analysis..docx]

Additional file1. Sequence of the primers used in qRT-PCR analysis.

| Gene | Forward Primer | Reverse Primer | Amplicon Length(bp) |
| --- | --- | --- | --- |
| LncRNA7695 | AACGGAGTAGATTGTGG | GTAGTTTAGTTGGAGCA | 139 |
| LncRNA4073 | GTAGTTTAGTTGGAGCA | TTCACATCCACCATTCC | 121 |
| LncRNA7410 | ATCGTTGTGTCCGTAGA | TGCTTGAGTGGTTTGTT | 102 |
| LncRNA9315 | CGGGAAGTTTGGTGTGT | CTGCGAGAGTGAGGGAC | 115 |
| LncRNA119 | GGGAAAGAAGAGATGGA | GGATGAGAAAGAGTGGC | 126 |
| LncRNA2234 | TCCCACCCTCCACCTCC | TGAACACCCGCACCAAA | 137 |
| LncRNA9628 | TCGCCGTCCTCTTCGTC | AAGCGGGGGTGTCCTCC | 98 |
| LncRNA3903 | TCTCTCTCCTCCCTGTA | TCATCTCTCAATCGTCA | 106 |
| LncRNA7092 | TGGGACGAGGCTCTTTC | AGTTCTTGAGGTGGCGG | 113 |
| LncRNA1366 | TTAGTATGGGGGTGGAG | CAAAAGTAAAAGGCGTG | 122 |
| UBQ | TGGTGTGAACGAGAAGGAAT | CCCTCAACAATCCCAAACC | 108 |
